# Advanced DNA Detection *via* Multispectral Plasmonic Metasurfaces

**DOI:** 10.3389/fbioe.2021.666121

**Published:** 2021-05-12

**Authors:** Valentina Di Meo, Massimo Moccia, Gennaro Sanità, Alessio Crescitelli, Annalisa Lamberti, Vincenzo Galdi, Ivo Rendina, Emanuela Esposito

**Affiliations:** ^1^Institute of Applied Sciences and Intelligent Systems Unit of Naples, National Research Council, Naples, Italy; ^2^Fields and Waves Laboratory, Department of Engineering, University of Sannio, Benevento, Italy; ^3^Department of Molecular Medicine and Medical Biotechnology, University of Naples Federico II, Naples, Italy

**Keywords:** plasmonic metasurface, localized surface plasmon resonances, surface enhanced infrared absorption spectroscopy, DNA biosensor, multiwavelength detection

## Abstract

We propose and demonstrate a sensing platform based on plasmonic metasurfaces for the detection of very low concentrations of deoxyribo-nucleic acid (DNA) fragments. The platform relies on surface-enhanced infrared absorption spectroscopy, implemented *via* a multispectral metasurface. Specifically, different regions (“pixels”) are engineered so as to separately cover the medium-infrared range of the electromagnetic spectrum extending from the functional-groups to the fingerprint region of a single analyte. In conjunction with a suitable bio-functionalization, this enables univocal and label-free recognition of specific molecules. For experimental validation, we fabricate a large-area gold metasurface on a silicon chip, and functionalize it with a recognition layer of peptide nucleic acid (PNA). Our experimental results indicate the possibility to detect complementary DNA fragments in concentrations as low as 50 fM, i.e., well below the value attained by standard methods, with additional advantages in terms of processing time, versatility and ease of implementation/operation.

## Introduction

For the early-stage diagnostic and therapy of genetic diseases, a precise identification of deoxyribo-nucleic acid (DNA) specific sequences is of paramount importance (Dwivedi et al., 2017). Such specificity in DNA sequences detection is also essential in applications requiring viral and bacterial investigation (López et al., 2003). Currently, standard methods (such as polymerase chain reaction, PCR technique) for DNA laboratory analysis are based on DNA amplification, which requires proper experimental design and control to achieve satisfactory sensitivities (Yang and Rothman, 2004). These methods, however, are time- and reagent-consuming, with a limit of detection (LOD) restricted to few nM (Liu et al., 2014; Fakih et al., 2017; Zhang et al., 2019). Therefore, they are not suited for wide-scale DNA testing, which requires instead high sensitivities, as well as small, fast and easy-to-use devices.

Within this framework, infrared (IR) spectroscopy constitutes a potentially attractive candidate, since it allows the univocal and label-free acquisition of direct information on molecules and their functional groups. In essence, IR radiation with wavenumbers within the range 10,000–100 cm^–1^ is absorbed by organic molecules, and then converted into variations of their rotational energy. In particular, due to the possibility to perform high-speed and standard investigations of several compounds, Fourier-transform IR (FTIR) spectroscopy is one of the most employed analysis tools for a wide range of scenarios of both academic and industrial interest (Stuart, 2004; Griffiths et al., 2007). The spectral range of greatest interest (4,000–400 cm^–1^) includes the so-called regions of “functional groups” (4,000–1,300 cm^–1^) and “fingerprint” (1,300–900 cm^–1^) (Chalmers and Griffiths, 2002). If there is no absorption in the former region, functional groups are absent in the analyzed molecule, whereas the bands appearing within the fingerprint region identify univocal features of the target analyte. However, as predicted by the Beer-Lambert law, the absorption signals become prohibitively weak in the presence of monolayers with minute amounts of analyte (and hence small IR absorption cross-section), which severely curtails the sensitivity of this technique (Amenabar et al., 2013). To overcome this limitation, it is of paramount importance to strongly enhance the light-matter interaction at molecular sites. In surface-enhanced IR absorption (SEIRA) spectroscopy (Le et al., 2008; Adato et al., 2015; Neubrech et al., 2017), this is attained by means of artificial materials such as plasmonic metasurfaces, i.e., 2-D arrays of sub-wavelength metallic inclusions (nanoantennas) (Chen et al., 2016). The functionalization of the metasurfaces is required to specifically capture the target analyte, while an accurate design of the nanoantennas is indispensable to support localized surface plasmon resonances with strong field enhancement (Neubrech et al., 2008; Adato et al., 2011; Di Meo et al., 2019). The optimal sensing conditions for SEIRA occur when the resonance spectrum matches the vibrational signature of the target molecule, so that it is modulated by the absorption phenomenon, thereby allowing its recognition (Adato et al., 2015; Neubrech et al., 2017). It is therefore essential to precisely tune the plasmonic resonances within the spectral range of interest, which can be attained by suitably dimensioning the nanoantenna geometrical parameters (shape, size, thickness and periodicity) (Novotny, 2007). From the fabrication viewpoint, this can be implemented with high spatial reproducibility (Aksu et al., 2010), and the SEIRA sensitivity can be optimized to detect a variety of chemical and biological analytes (Adato and Altug, 2013; Di Meo et al., 2020).

In this study, we report a label-free and real-time detection of DNA fragments by means of SEIRA spectroscopy based on multispectral plasmonic metasurfaces. The proposed device is a large-area (several mm^2^) metasurface organized in “pixels,” consisting of different spatial regions engineered so as to work within separate IR spectral regions of interest (spanning from the fingerprint region to the region of the functional groups). Pixels are constituted by 2D arrays of cross-shaped gold nanoantennas, realized on a silicon substrate by means of electron beam lithography (EBL), which ensures a reproducible fabrication of homogeneous and robust devices (Di Meo et al., 2019). The plasmonic response of each array (arising from both the collective and individual response of the nanoantennas) generates enhancement factors up to 10^6^. Furthermore, the symmetric shape of the nanoantennas minimizes the sensitivity with respect to the polarization of the impinging radiation and to the random orientation of the target analyte dipole moment.

In order to detect DNA fragments, we exploit a peptide nucleic acid (PNA) as a recognition layer, since its IR spectrum is different from that of DNA. Moreover, due to its properties, PNA exhibits a higher affinity and specificity binding capabilities to complementary nucleic acids with respect to traditional oligonucleotides (Wittung et al., 1994), even higher than the corresponding ssDNA sequence (Wang, 1998). For these reasons, PNA is one of the most widely employed tools for the development of innovative biosensors. We are able to detect DNA fragments at a concentration as low as 50 fM, therefore much lower than traditional methods, in a label-free and real-time modality. These results demonstrate the very promising potential of the proposed approach, which is non-destructive, fast, cost-effective, and based on miniaturized devices. As a more general outcome, our study indicates that the knowledge of the IR absorption properties of a given molecule can be effectively used to detect its presence, providing the molecular fingerprint at extremely low concentrations.

## Materials and Methods

### Modeling and Design

**FIGURE 1:**
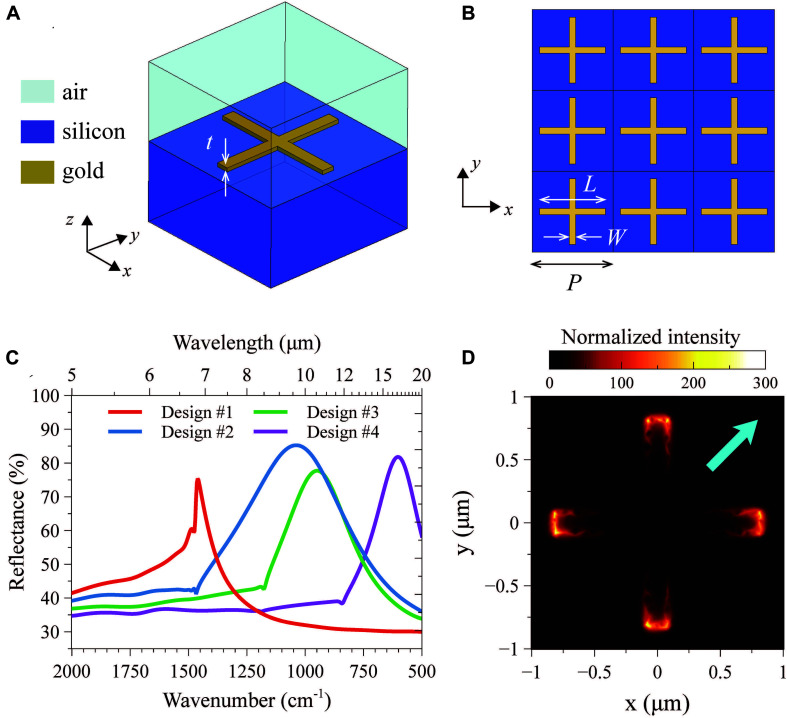
**(A,B)** 3-D schematic of a single unit-cell and top view of the periodic array, respectively, with indication of the associated reference systems, materials, and geometrical parameters. **(C)** Numerically computed reflectance spectra, for a normally incident plane-wave illumination with *x*-polarized electric field, pertaining to the four selected designs (with parameters given in Table 1). **(D)** Electric-field intensity distribution (normalized with respect to the impinging one, in false-color scale) over the unit cell, for design #2, computed at a distance of 10 nm (along *z*) from the nanoantenna at the resonance (wavenumber: 1041*cm*^−1^). For better visualization, the impinging electric field is chosen as not aligned to the cross arms (see the cyan thick arrow).

The plasmonic metasurfaces in our study are modeled and designed by means of finite-element numerical simulations carried out *via* the commercial software package Ansys HFSS electromagnetics suite 16.2^1^. Referring to Figures 1A,B for a schematic illustration, we assume a silicon substrate [with refractive index taken from (Chandler-Horowitz and Amirtharaj, 2005)] overlayed with a 2-D periodic arrangement of cross-shaped gold nanoantennas of thickness *t=50*nm, period *P*, arm-length *L*, arm-width *W*, and electrical conductivity σ = 15×10^6^ S/m (Laman and Grischkowsky, 2008), with an exterior air region.

The structure, assumed of infinite extent in the transverse plane, is modeled considering a single period (unit cell, Figure 1A), under plane-wave illumination normally impinging from the air region. Accordingly, master/slave periodicity conditions are enforced at the lateral walls, whereas a Floquet-type port and a perfectly matched layer are assumed as terminations of air region (of thickness 10 μm) and substrate (of thickness 5 μm), respectively. The computational domain is finally discretized *via* standard meshing (resulting in ∼160,000 elements) and the problem is numerically solved by means of the standard iterative solver with default converge conditions.

Based on an extensive set of parametric studies, we identify the resonance regimes and investigate their dependence on the geometrical parameters; this enables the tuning of the resonant lineshapes within the spectral region of interest. Figure 1C shows the numerically simulated reflectance spectra pertaining to the four design selected (with geometrical and resonance details summarized in Table 1). We also observe some abrupt changes of slopes at specific wavenumbers, which are due to the well-known Rayleigh-Wood anomalies for the transmitted field (Maystre, 2012). A representative resonant-field distribution is shown in Figure 1D as a normalized intensity map (in false-color scale) over a single unit cell. As typical for plasmonic nanoantennas, we observe that the field is strongly enhanced (up to a factor ∼300) at the arm tips, yielding very pronounced hotspots.

**TABLE 1:** Nominal geometrical parameters for the four designs in Figure 1C, with indication of the corresponding (simulated) resonance wavenumbers.

**Pixel**	***L* (μm)**	***W* (μm)**	***P* (μm)**	**$\nu_{\mathrm{res}}^{\mathrm{sim}}$ (cm^–1^)**
#1	1.1	0.2	2.0	1,461
#2	1.7	0.2	2.0	1,041
#3	2.0	0.2	2.5	947
#4	3.0	0.2	3.5	604

### Fabrication Methods

**FIGURE 2:**
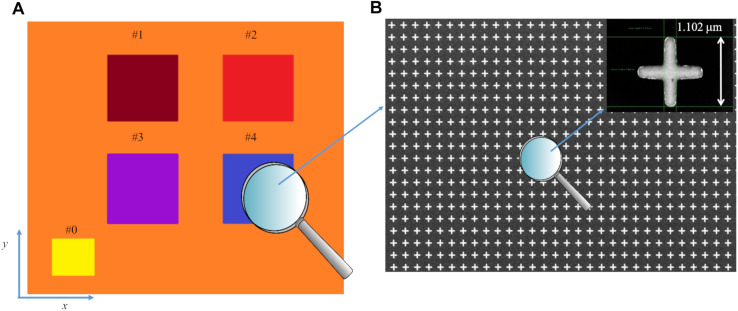
**(A)** schematic of the multiplexed metasurface. Each pixel is identified by an index (from #1 to #4), with #0 indicating the plain-gold reference pixel for FTIR measurements. **(B)** SEM image of a representative pixel, with a magnified view of a single nanoantenna.

The multispectral metasurfaces are fabricated on a 1 cm^2^ silicon wafer die, with the coverage area spanning over 1.5 mm × 1.5 mm. As schematically illustrated in Figure 2A, the metasurface comprises four different pixels, according to the designs in Figure 1C and Table 1. In particular, the resonance wavenumbers span the range 600–1,500 cm^–1^, in order to include the fingerprint region. Each pixel is identified by its coordinates on the die (with respect to a fixed Cartesian system), and covers an area of 500 μm × 500 μm, which is well matched with the typical 100 μm × 100 μm beam-width of the FTIR spectrometer. The fabrication relies on the EBL technique, whereby each nanoantenna is first patterned in electron resist, and the design is then transferred into a 50 nm gold film *via* a lift-off process (Crescitelli et al., 2012). Figure 2B shows a scanning electron microscope (SEM) image of a representative pixel, with a magnified view of a single nanoantenna. The standard method utilized guarantees a very high reliability level, and the fabricated devices are reusable. All the details on the fabrication process and related data are reported in the supplementary information of (Di Meo et al., 2019), and are not repeated here.

### Functionalization Procedure

**FIGURE 3:**
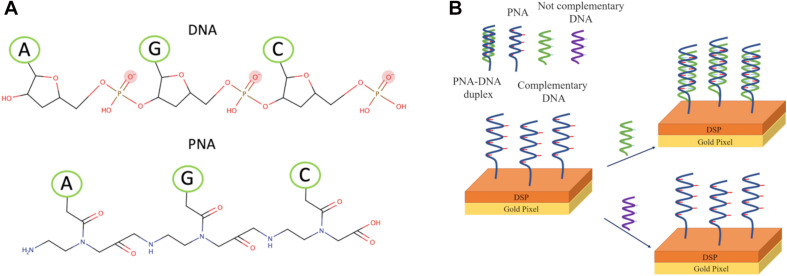
**(A)** Molecular structure of ssPNA and ssDNA. **(B)** Schematic of binding ssPNA with ssDNA.

To detect the capture of DNA on the metasurface pixels, we use a single-stranded PNA (ssPNA) as recognition layer that supports the DNA base pairing mechanisms. Indeed, PNA emulates structural DNA by substituting its typical ribose phosphate backbone with N-(2-aminoethyl) glycine linkage. As shown in Figure 3A, methylene carbonyl linkers bind the nitrogenous bases of PNA to the polyamide structure (Nielsen et al., 1991; Egholm et al., 1992).

All pixels are bio-functionalized with *RAS*-PNA-NH_2_ by using dithiobis (succinimidyl propionate) (DSP) as linker. In particular, the pixels are washed with dimethyl sulfoxide (DMSO), isopropyl alcohol, and bi-distilled water for 5′ under stirring. After drying by N_2_ flux, the gold surface is incubated with DSP 4 mg/ml in DMSO for 60′ at room temperature. After extensive washing with DMSO, bi-distilled water and phosphate buffered saline (PBS) solution 1×, the surface is incubated with *RAS*-PNA-NH_2_ (molecular weight, MW = 3,714 Da) 80 μM in PBS over night at 4°C. Next, after extensive washing with PBS 1× and bi-distilled water, the pixels are dried by N_2_ fluxed, and incubated with complementary DNA (cDNA) (MW = 3,572 Da) at 50 fM, 500 fM and 5 pM, or with not complementary DNA (ncDNA) (MW = 3,748 Da) at 50 pM in PBS 1× for 6 h at 4°C. After washing with PBS 1× and bi-distilled water to remove the unbounded DNA, the pixels are dried with N_2_ flux. Binding measurements are carried out *via* the procedure described above. A scheme of the binding process is shown in Figure 3B.

### IR Measurements

The reflectance spectra are acquired by means of a Thermo-Nicholet NEXUS Continuum XL (Thermo Scientific, Waltham, MA, United States) spectrometer equipped with a nitrogen cooled mercury cadmium telluride detector and a focal-plane-array detector. Knife-edge apertures are set to delimit a measurement area of 100 × 100 μm^2^. Measurements are carried out at room temperature on dried samples, by collecting 200 scans with a resolution of 4 cm^–1^ within the range 4,000–600 cm^–1^. All acquired spectra are normalized with respect to a background that is collected on a plain gold film deposited on the same substrate of the multispectral metasurface (pixel #0 in Figure 2A).

## Results and Discussion

**FIGURE 4:**
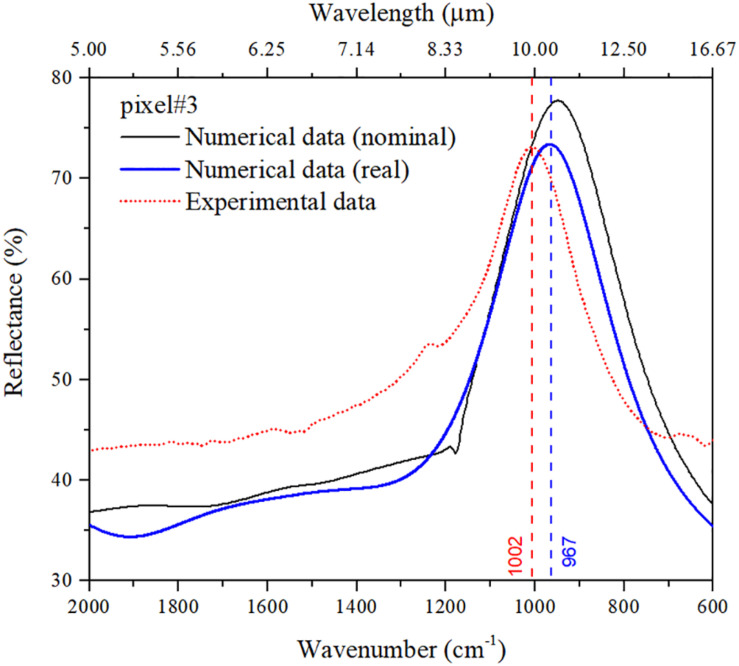
Comparison between numerical and experimental reflectance curves pertaining to pixel #3 for naked nanoantennas (no molecules adsorbed). Besides the prediction for the nominal design, also shown is the numerical spectrum computed by assuming the actual dimensions (*L* = 1.94μ*m*,*P* = 2.5μ*m*,*W* = 0.155μ*m*) estimated from the SEM image.

Figure 4 shows a comparison between simulated and measured reflectance spectra of a representative pixel. In this particular example, pixel #3 exhibits a plasmonic resonance at a nominal wavenumber of 947 cm^–1^ with ∼75% reflectance. As can be observed, the position of the experimental resonance peak is in fair agreement with the numerical prediction for the nominal design (see Table 1). The discrepancies are mostly attributable to fabrication tolerances, and indeed the agreement improves if the actual nanoantenna dimensions estimated from the SEM images are considered in the simulations (see blue curve). Overall, our design procedure of the metasurface pixels turns out to provide sufficient accuracy for tuning the desired spectral characteristics.

**FIGURE 5:**
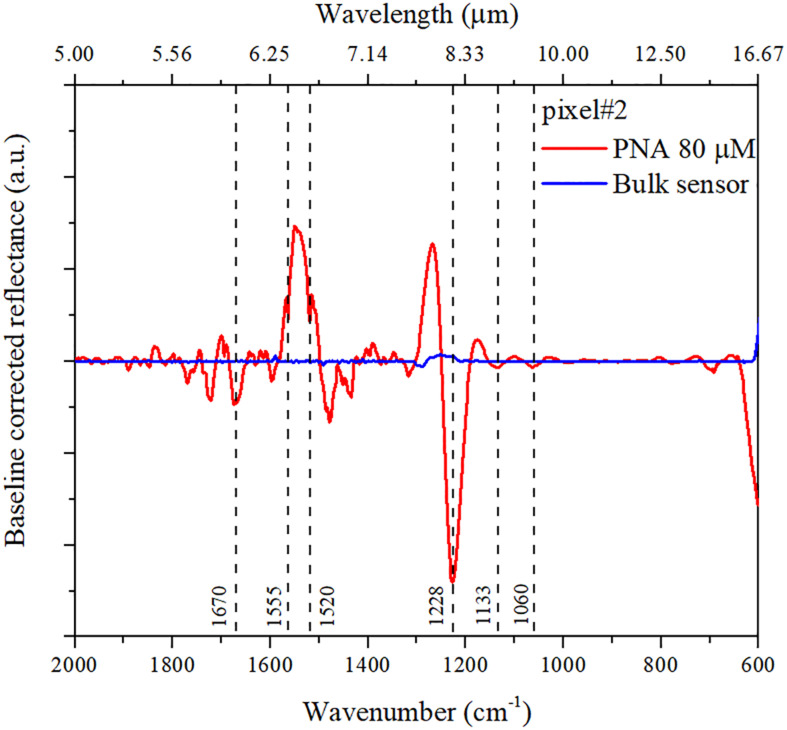
SEIRA baseline-corrected reflectance spectra pertaining to pixel #2. The blue curve pertains to the pixel without functionalization (i.e., naked nanoantennas); the red curve pertains to nanoantennas with PNA molecules linked, with the detected molecular vibrations as described into the text.

As previously mentioned, the specific detection of DNA sequences is fundamental for the diagnostic and treatment of genetic-related diseases (Yang et al., 1997; Dwivedi et al., 2017). To detect the capture of DNA on the metasurface pixels, we exploit PNA as a recognition layer that supports the DNA base pairing mechanisms (Wang, 1998). The reference analytical characterization used in our experiment, based on the FTIR analysis of solid samples of *RAS*-PNA-NH2 and complementary DNA (1.68 μM each), is provided in the Supplementary Material (Supplementary Figure 1). Precisely by virtue of the chemical differences between DNA and PNA, we are able to recognize the DNA binding on our nanoantennas through the signature of the vibrational band of the sugar-phosphate backbone (1,000–1,300 cm^–1^) (more specifically, *via* the asymmetric stretching vibration of PO_4_^–^ group). The gold nanoantennas are functionalized with a *RAS*-PNA recognition layer, and the FTIR spectra are examined before and after the PNA binding on their surface, and once again after the binding with the DNA. The absolute amount of PNA molecules linked on the whole chip area of 1 cm^2^ is estimated to be 1.60 nmol (a droplet of 20 μL at a concentration of 80 μM is adsorbed). Considering an effective area coverage exposed to the light of 100 × 100 μm^2^, we are able to detect in a single measurement an absolute amount of 160 fmol of PNA. Figure 5 shows the SEIRA baseline-corrected reflectance spectra pertaining to pixel #2 (red curve) after the binding of PNA molecules, and the FTIR baseline-corrected reflectance spectra of the same pixel without functionalization (blue curve) as a negative control. Pixel #2 is characterized by a naked resonance nominally centered at 1,041 cm^–1^.

**FIGURE 6:**
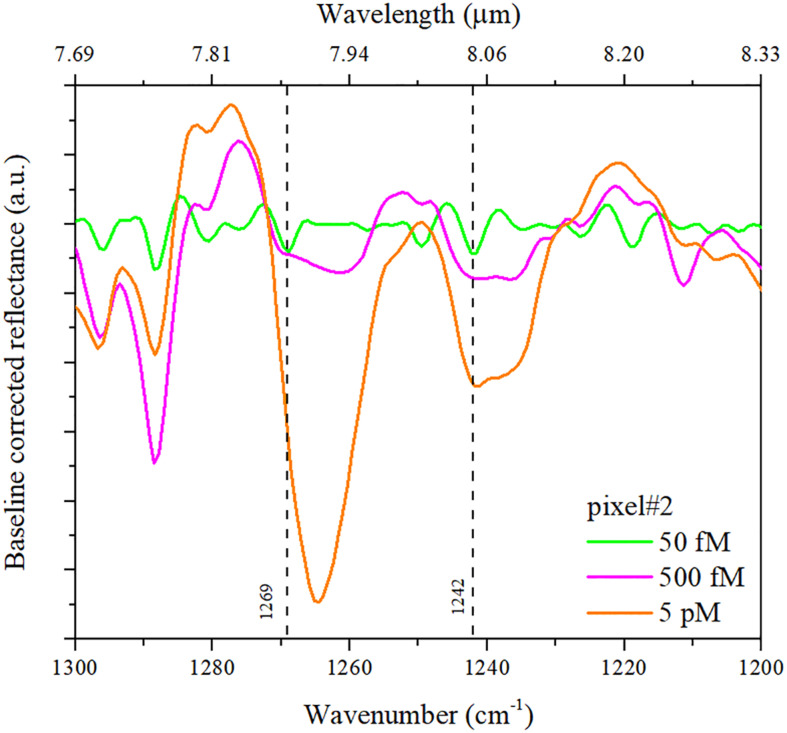
SEIRA baseline-corrected reflectance spectra pertaining to pixel #2 at different concentrations of cDNA: 50 fM (green curve), 500 fM (purple), and 5 pM (orange).

The SEIRA spectra pertaining to pixel #2 show the signature of PNA, as deduced from a comparison with the spectrum pertaining to a solid sample of the same PNA molecules utilized in the experiment (see Supplementary Figure 1). In particular, the bending vibrations [δ(C-H) and δ(N-H)] and in-plane vibration mode (of C-C and C = N) (Wang et al., 2001; Yamada et al., 2004) of adenine and cytosine nitrogenous bases appear around 1,550 cm^–1^. Other vibrations that appear in the same region are related to the amide group: Amide I around 1,650 cm^–1^ (C = O stretch) and Amide II at 1,540 cm^–1^ (NH deformation). These frequencies are difficult to identify because they are mixed with the nucleic base absorptions and exhibit very low intensity (Mateo-Martí et al., 2005). Figure 6 shows the SEIRA baseline-corrected reflectance spectra pertaining to pixel #2 after the binding of the PNA recognition layer with the complementary DNA (cDNA) at different concentrations (from 50 fM to 5 pM).

Once the bond occurs between the PNA and DNA, the sugar-phosphate conformation is present. The SEIRA spectra in Figure 6 exhibit the vibrational band of the sugar-phosphate backbone (1,000–1,300 cm^–1^) (Banyay et al., 2003) (more specifically, the asymmetric stretching vibration of PO_4_^–^). The antisymmetric PO_2_^–^ stretching band is a characteristic marker for nucleic acid backbone conformation, independent of nitrogenous bases vibrations and sugar pucker. As the concentration is reduced to 50 fM, the intensity of the bands is reduced, but the signature remains well visible at 1,269 and 1,242 cm^–1^. However, since these bands are found within the fingerprint region, it is not always easy to assign them. In addition, the vibrational characteristics of the chemical groups of nucleic acid (especially PO_2_^–^) also depend on the DNA composition (Mello and Vidal, 2012).

**FIGURE 7:**
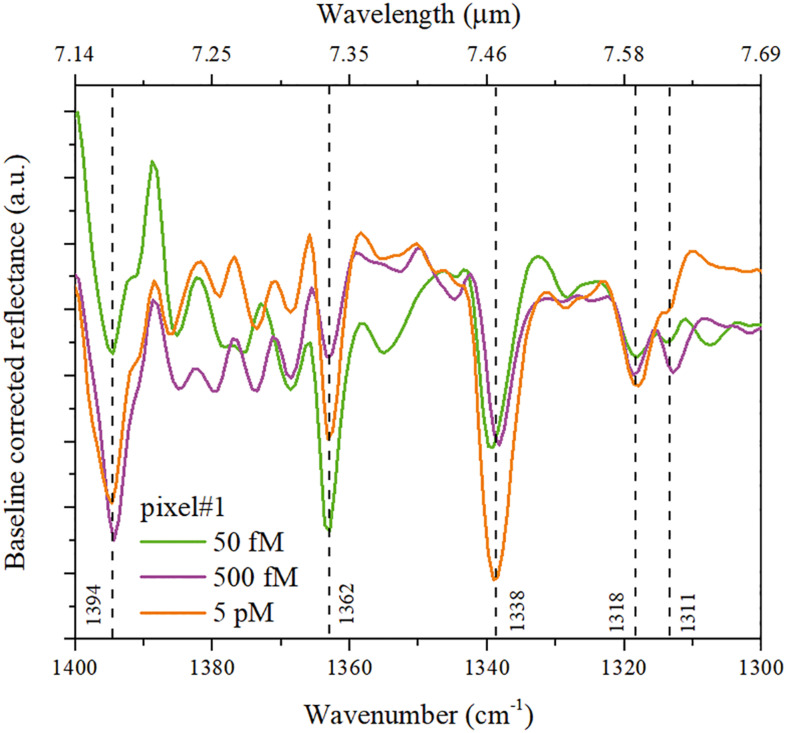
SEIRA baseline-corrected reflectance spectra pertaining to pixel #1 at different concentrations of cDNA: 50 fM (green curve), 500 fM (purple), and 5 pM (orange).

Pixel #1 is characterized by a naked resonance nominally centered at 1,461 cm^–1^. After the binding with the correlated DNA, it exhibits SEIRA vibrational bands, corresponding to base–sugar vibrations, within the region 1,394–1,311 cm^–1^. This region is sensitive to glycosidic bond rotation, backbone conformation and sugar pucker (Peng et al., 2011). The SEIRA baseline-corrected reflectance spectra pertaining to pixel #1 are shown in Figure 7.

To sum up, based on the reference IR analysis carried out on the DNA sample in solid form (Supplementary Figure 1), it is possible to associate all the peaks highlighted through the SEIRA effect to the corresponding vibrational bands of the molecule.

As previously mentioned, in order to attain a manageable vibrational signal, it is crucial to precisely tune the nanoantenna resonances within the different vibration bands. The sensitivity of resonant SEIRA effects can be quantified in terms of the enhancement factor (EF) (Neubrech et al., 2017).

(1) $$E\,F=\frac{\Delta \,R}{\Delta \,R_{0}}\cdot \frac{A_{0}}{A_{S\,E\,I\,R\,A}}$$

with Δ*R* denoting the reflectance difference (at the vibrational signature) in the absence and presence of the molecules, and Δ*R*_0_ the reflectance difference with respect to a flat gold layer with same thickness as the nanoantennas (pixel #0 in Figure 2A). Moreover, *A*_0_ indicates the effective area of the nanoantennas illuminated (100 μm × 100 μm, in our case), and *A*_SEIRA_ the effective area of the nanoantennas on which the field is localized. As well known (Neubrech et al., 2008; Adato et al., 2011; Di Meo et al., 2019, 2020), and also observed in Figure 1D, the resonant field tends to be mainly localized at the tips of the nanoantenna arms. As detailed in the Supplementary Material, we estimate EF values of 7 × 10^6^ for pixel #2, and 2.3 × 10^6^ for pixel #1, which turn out to be among the largest values reported in the literature (typically ranging from 10^3^ to 10^5^) (De Tommasi et al., 2021), thereby confirming the attractive performance of our devices and the very promising potential of the approach.

The minimum concentration of correlated DNA fragments detected in our experiment is 50 fM, which is well below the value (0.87 nM) determined by standard and innovative methods (Liu et al., 2014; Fakih et al., 2017; Zhou et al., 2018, 2019a,b; Zhang et al., 2019). The absolute amount of DNA molecules detected for this last concentration is 1 amol spread on a die area of 1 cm^2^, corresponding to an actual amount on the detection area of 100 × 100 μm^2^ in the zepto molar range. In a parallel control experiment, we also verified the specificity of the molecular recognition by exposing the sensor to a non-complementary DNA (ncDNA) at 50 pM. As shown in the Supplementary Material, in this case, we do not observe any difference in the reflectance spectra of our device before and after the linking procedure with the active layer of PNA (see Supplementary Figure 2), thereby confirming the failure of the binding procedure, and hence the selectivity of the proposed platform.

As shown in the Supplementary Material, the dynamic curve of our sensor exhibits a linear behavior, thereby suggesting the possibility to further lower the LOD (see Supplementary Figure 3). Although the range we have explored does not represent the full working range, we will plan further experiments close to the noise level in order to estimate the ultimate LOD. It is worth emphasizing that, besides the capability to detect extremely low concentrations of analyte, our proposed sensor based on multispectral metasurfaces compares favorably with traditional methods based on radioactive compounds (Wei et al., 2010), since it does not require long response times, large amount of reagents, disposal, pretreatment procedures, and expensive instrumentation.

## Conclusion

In this study, we have demonstrated a multispectral plasmonic metasurface sensor for the multiwavelength detection of ssDNA at very low concentration based on SEIRA spectroscopy. Unlike many traditional methods (based on radioactive compounds), our technology enables a label-free and real time detection, thus providing inherent advantages, including cost effectiveness. We have verified the DNA detection capability of our sensing platform using PNA molecules as a recognition layer that supports the DNA base pairing mechanisms, thereby ensuring selectivity and specificity. We have recognized the molecular fingerprint of the DNA through the identification of the specific vibrational bands in the SEIRA spectra. The measured LOD is 50 fM, which corresponds to an absolute amount of 1 amol of DNA molecules over the whole die area, which is significantly lower than the values characterizing both traditional and innovative approaches proposed so far. Overall, the obtained results indicate promising perspectives for the technology underpinning our biosensor. It can be extended to the detection of viruses and other pathogens by functionalizing the sensing metasurface with suitable probes—like antibodies—able to specifically recognize target antigens expressed on the pathogens surface.

## Data Availability Statement

The original contributions presented in the study are included in the article/Supplementary Material, further inquiries can be directed to the corresponding author/s.

## Author Contributions

VDM manufactured the devices and analyzed the data. MM designed and simulated the devices. GS functionalized the devices and performed the trials using FTIR spectrometer. AC contributed to the manufacturing. AL supervised the functionalization and trials. VG supervised the design and modeling, and wrote the manuscript. IR supervised the device realization. EE conceived the idea, led the investigation, analyzed the data, and wrote the manuscript. All authors reviewed the manuscript.

## Conflict of Interest

The authors declare that the research was conducted in the absence of any commercial or financial relationships that could be construed as a potential conflict of interest.

## Footnotes

1 https://www.ansys.com/products/electronics/ansys-hfss
