# Supplementary material for: Advanced DNA Detection via Multispectral Plasmonic Metasurfaces
Source: Front Bioeng Biotechnol. 2021 May 12;9:666121. doi: 10.3389/fbioe.2021.666121 (PMC8149789; doi:10.3389/fbioe.2021.666121)
Supplement: Supplementary file 1 [file Data_Sheet_1.DOCX]

Supplementary Material

**1 Details on the EF Estimation**

In the expression of the EF, given in Eq. (1) of the main text, *A_SEIRA_* indicates the effective nanoantenna area on which the field is localized. As reported in the literature (Coates, 2006), and also apparent in **Figure 1D** of the main text, such localization essentially occurs at the tips of the nanoantenna arms. Accordingly, the active area for each nanoantenna can be estimated as the sum of four semi-circles with a diameter *W*=200 nm, localized at the tips of cross arms, i.e., *A_NA_* ≈6.28×10^4^ nm^2^. Taking into account the exposed area coverage, we obtain *A_SEIRA_* = 5×10^7^ nm^2^ for pixel #2 and *A_SEIRA_* =10^8^ nm^2^ for pixel #1. Recalling that the area of the nanoantennas exposed to the IR radiation is *A*_0_=10^10^ nm^2^, this yields a ratio *A*_0_/*A_SEIRA_* (also called “geometrical enhancement factor”) of 200 and 100, respectively. This geometrical value is multiplied by the gain factor ΔR/ΔR_0_, which depends on the enhanced signal. Specifically, we have ΔR_0_ = 0.001%, while ΔR is 35% for pixel #2 and 23% for pixel #1.

In conclusion, we estimate an EF of 7×10^6^ for pixel#2, and 2.3×10^6^ for pixel#1.

**2 Reference FTIR Characterization**

**Supplementary Figure 1** shows the reference analytical characterization, in terms of FTIR absorbance spectra of solid samples of *RAS*-PNA-NH2 and complementary DNA.

**

**

**Supplementary Figure 1.** Reference FTIR characterization of a solid sample of 1.68 μM PNA and DNA.

**3 Not complementary DNA Measurements**

In order to assess the specificity of the proposed sensor, we also test the binding on the metasurface of a not complementary DNA at a concentration of 50 pM, i.e., an order of magnitude larger than the one considered for complementary DNA. The failure of the binding process can be observed in **Supplementary Figure 2**, which compares the baseline-corrected spectra pertaining to pixel #2 in the presence of complementary (cDNA) and not complementary (ncDNA) DNA.

**

**

**Supplementary Figure 2.** SEIRA baseline-corrected spectra pertaining to pixel #2, for cDNA and ncDNA, after the binding procedure.

**4 Dynamic Curve**

In order to estimate the LOD of our device, we consider the dynamic curve pertaining to pixel #2. **Supplementary Figure 3** shows the intensity of the baseline corrected-signals within the band 1242-1235 cm^-1^ as a function of the DNA concentration. The curve exhibits a good linearity, suggesting the possibility to detect DNA concentrations even lower than 50 fM.

**

**

**Supplementary Figure 3.** Dynamical curve pertaining to pixel #2.

**References**

Coates, J. (2006). “Interpretation of Infrared Spectra, A Practical Approach,” in *Encyclopedia of Analytical Chemistry* (American Cancer Society). doi:10.1002/9780470027318.a5606.
